# Radial self-navigated native magnetic resonance angiography in comparison to navigator-gated contrast-enhanced MRA of the entire thoracic aorta in an aortic patient collective

**DOI:** 10.1186/s12968-021-00774-9

**Published:** 2021-07-12

**Authors:** Martina Correa Londono, Nino Trussardi, Verena C. Obmann, Davide Piccini, Michael Ith, Hendrik von Tengg-Kobligk, Bernd Jung

**Affiliations:** 1 Department of Diagnostic, Interventional and Pediatric Radiology (DIPR), Inselspital, Bern University Hospital, University of Bern, Freiburgstrasse 10, 3010 Bern, Switzerland; 2 Department of Radiology, University Hospitals Cleveland Medical Center, Case Western Reserve University, Cleveland, OH USA; 3 Advanced Clinical Imaging Technology, Siemens Healthcare AG, Lausanne, Switzerland; 4 Department of Diagnostic and Interventional Radiology, Lausanne University Hospital and University of Lausanne, Lausanne, Switzerland; 5 Experimental Radiology, Department of BioMedical Research, University of Bern, Bern, Switzerland

**Keywords:** Steady-state free precession MRA, MRI angiography, Thoracic aorta, Aortic diseases

## Abstract

**Background:**

The native balanced steady state with free precession (bSSFP) magnetic resonance angiography (MRA) technique has been shown to provide high diagnostic image quality for thoracic aortic disease. This study compares a 3D radial respiratory self-navigated native MRA (native-SN-MRA) based on a bSSFP sequence with conventional Cartesian, 3D, contrast-enhanced MRA (CE-MRA) with navigator-gated respiration control for image quality of the entire thoracic aorta.

**Methods:**

Thirty-one aortic native-SN-MRA were compared retrospectively (63.9 ± 10.3 years) to 61 CE-MRA (63.1 ± 11.7 years) serving as a reference standard. Image quality was evaluated at the aortic root/ascending aorta, aortic arch and descending aorta. Scan time was recorded. In 10 patients with both MRA sequences, aortic pathologies were evaluated and normal and pathologic aortic diameters were measured. The influence of artifacts on image quality was analyzed.

**Results:**

Compared to the overall image quality of CE-MRA, the overall image quality of native-SN-MRA was superior for all segments analyzed (aortic root/ascending, p < 0.001; arch, p < 0.001, and descending, p = 0.005). Regarding artifacts, the image quality of native-SN-MRA remained superior at the aortic root/ascending aorta and aortic arch before and after correction for confounders of surgical material (i.e., susceptibility-related artifacts) (p = 0.008 both) suggesting a benefit in terms of motion artifacts. Native-SN-MRA showed a trend towards superior intraindividual image quality, but without statistical significance. Intraindividually, the sensitivity and specificity for the detection of aortic disease were 100% for native-SN-MRA. Aortic diameters did not show a significant difference (p = 0.899). The scan time of the native-SN-MRA was significantly reduced, with a mean of 05:56 ± 01:32 min vs. 08:51 ± 02:57 min in the CE-MRA (p < 0.001).

**Conclusions:**

Superior image quality of the entire thoracic aorta, also regarding artifacts, can be achieved with native-SN-MRA, especially in motion prone segments, in addition to a shorter acquisition time.

## Background

Magnetic resonance angiography (MRA) has been established for serial follow up studies of patients with known aortic disease [1], with the application of contrast media as a reference technique [1–3]. However, previous studies demonstrated the usefulness of native balanced steady-state free precession (bSSFP) acquisition techniques for obtaining high diagnostic accuracy and image quality of the thoracic aorta [4, 5].

To avoid artifacts due to cardiac motion, data acquisition is typically synchronized with the electrocardiogram (ECG). Respiratory motion is commonly controlled by navigator gating [6] with known disadvantages of reported low acceptance rates of less than 40% in patients with irregularities of the respiratory pattern [7, 8] resulting in unpredictable long scan times.

Recently, a 3D radial self-navigated (SN) method based on a bSSFP sequence was introduced for imaging the coronary arteries and the entire aorta, including iliofemoral run-off, without the need for contrast media [9–11]. SN is based on a signal from the imaging data itself that is used for respiratory motion correction [9, 12, 13]. With its radial trajectory for data acquisition, it is less prone to motion artifacts than conventional Cartesian data acquisition [14, 15]. A second aspect is the data acquisition efficiency of 100%, resulting in a defined scan time independent of patient respiratory patterns, simplifying examination planning [9].

The purpose of this study was to compare a native 3D radial SN MRA sequence (native-SN-MRA) with a Cartesian 3D contrast enhanced (CE)-MRA sequence as the gold standard for the evaluation of thoracic aortic diseases in terms of image quality parameters in an aortic patient collective.

## Materials and methods

### Patients

**Fig. 1:**
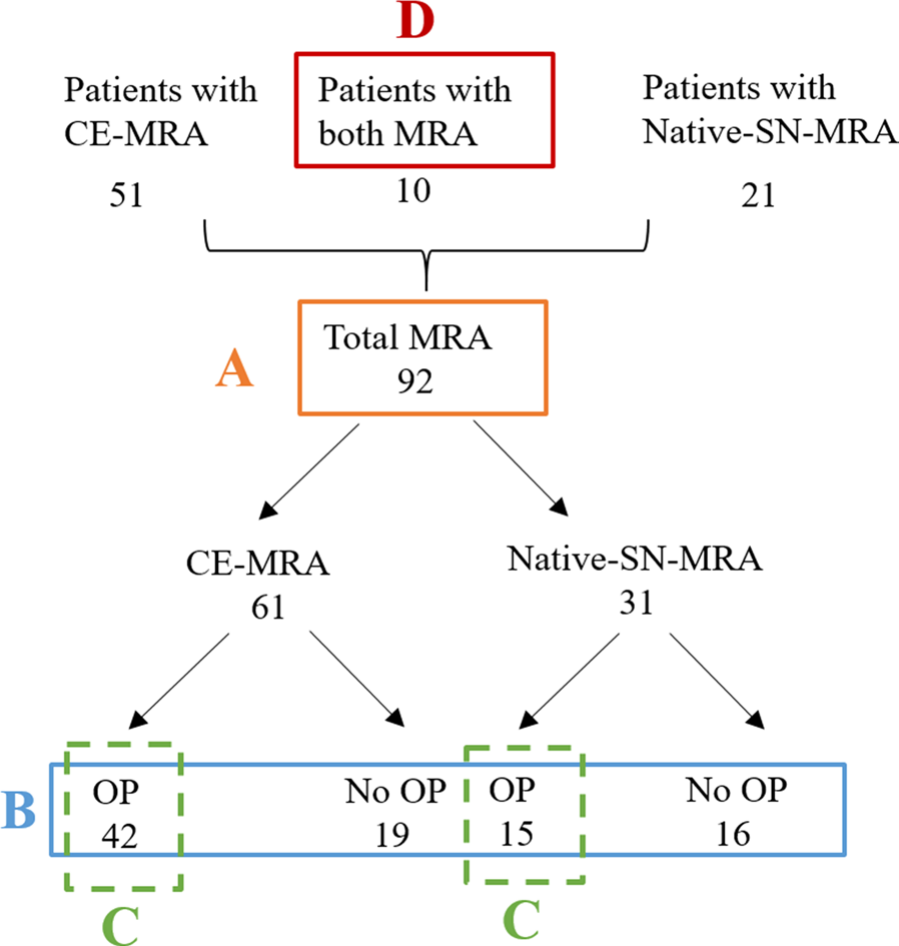
Study flow chart. **A** Overall image quality was tested for statistical significance in 31 native-self-navigated (SN)-MRA and 61 contrast enhanced (CE)-MRA with binary and 4-point scale ratings (orange). **B** Analysis of the influence of artifacts on both sequences (blue). **C** Subgroup analysis of patients following aortic surgery only (green). **D** In the intraindividual comparison of 10 patients with both types of MRA the overall image quality was tested (red). *CE-MRA* contrast-enhanced magnetic resonance angiography, *native-SN-MRA* 3D radial respiratory self-navigated non-contrast-enhanced MRA, *OP* operation

The study was approved by the local institutional review board (KEK-Nr. 2018-01596). Eighty-two patients with 92 thoracic aortic MRAs from our institution were identified in this retrospective cross sectional, single center study. Only patients who received gadobenate dimeglumine or gadoterate meglumine contrast agents were included. A total of 61 high resolution CE-MRA between April 2017 and May 2018 and 31 native-SN-MRA from March 2015 to May 2018 were available for analysis. All 82 patients received the contrast agent on which diagnosis was based. Only 10 patients with native-SN-MRA received one of the two included contrast agents; therefore, only in these patients were both MRA sequences performed (Fig. 1). In the remaining 21 patients with native-SN-MRA an intravascular contrast agent which did not meet the inclusion criteria was used.

The mean patient age was 63.4 ± 11.0 years (range 32 to 84 years) including  67 men [63.1 ± 10.9 years, range, 32 to 84 years] and 25 women [65.5 ± 11.6 years; range, 46 to 84 years]). The mean body weight was 79.4 ± 14.3 kg, and the mean patient height was 171.9 cm ± 9.4 cm. There was no significant difference in age, body weight or height between the two MRA groups (p = 0.76, 0.07, and 0.33, respectively; the Wilcoxon rank sum test).

An MRA examination, including the application of contrast media, is frequently a part of clinical follow-up after surgery of the aortic root up to and often including the aortic arch. As part of this procedure, surgical materials such as sternal cerclages, aortic valve replacements, and vascular clips additionally challenge MRA image quality.

In this study, thoracic aortic diseases included known aortic dissection (n = 70), intramural hematoma (n = 2), penetrating aortic ulcer (n = 1) and thoracic aorta aneurysm (n = 9). For all studies, CE-MRA was considered the gold standard.

### Image acquisition

All examinations were performed on a 1.5 T CMR scanner (Magnetom Aera, Siemens Healthineers, Erlangen, Germany). The imaging protocol consisted of the following major sequences (Table 1):

**Table 1:** MRA sequence parameters

Sequence parameter	Native-SN-MRA	CE-MRA
Sequence type	bSSFP (TrueFISP)	T1-GRE
Acquisition trajectory	Radial	Cartesian
Respiration control	Self-navigated	Navigator based
Data efficiency	100%	Respiration dependend (ca. 50%)
TR	3.6 ms	3.4 ms
TE	1.83 ms	1.33 ms
Radial undersampling factor	5	n.a
Fat saturation	Spectral	Spectral
FOV	Isotropic, 250 mm	340 × 255 × 83 mm
Resolution	Isotropic, 1.3 mm	1.3 × 1.3 × 1.4 mm
Contrast media	No	Yes

*CE-MRA* contrast-enhanced MRA, *Native-SN-MRA* 3D radial respiratory self-navigated non-contrast-enhanced MRA, *TE* echo time, *TR* repetition time, *FOV* field of view, *bSSFP* balanced steady state with free precession, *GRE* gradient echo

In 31/92 of thoracic aortic MRA examinations, a native sequence based on an ECG-triggered SN prototype 3D radial bSSFP sequence (TE = 1.83 ms; TR = 3.6 ms, radial undersampling factor of 5) with T2-prep, spectral fat saturation and an acquisition window of 128 ms during the quiescent phase within the cardiac cycle was acquired with an inherent isotropic FOV of 250 mm and spatial resolution of 1.3 mm [9].

In 61/92 of the thoracic aortic MRA examinations, an ECG-triggered 3D CE-MRA (TE = 1.33 ms; TR = 3.4 ms) with navigator respiration control, T2-prep, spectral fat saturation and an acquisition window of 124 ms during the quiescent phase within the cardiac cycle was acquired of the entire thoracic aorta with an FOV of 340 × 255 × 83 mm, a spatial resolution of 1.4 × 1.3 × 1.3 mm, a width of 7–8 mm and 0.1 ml/kg body weight gadobenate dimeglumine at a flow rate of 0.4 ml/s.

We used navigator-gated CE-MRA, especially to yield a high 3D spatial resolution of segments prone to cardiac motion, namely the aortic root and the ascending aorta, to be able to measure aortic diameters as accurately as possible. In addition, compared to first-pass standard-of-care MRA, navigator-gated CE-MRA is less operator dependent. Because ECG synchronization and respiration control are required in CE-MRA, the scan time was in the range of minutes rather than approximately 20 s. Therefore, the flow rate of the contrast agent was chosen to be rather low in comparison to a “conventional” first-pass MRA with the breath-hold technique. To compensate for this issue, centric k-space reordering was chosen to ensure that the image contrast is mostly acquired during the arterial passage and therefore to minimize the venous signal in CE-MRA. Time-resolved 3D MRA was used to evaluate the bolus arrival time in the thoracic aorta in advance.

## Evaluation of MRA images

### Qualitative analysis

The focus of the evaluation was image quality as defined by the delineation of the aortic wall and therefore to ensure a reliable measurement of the diameter of the thoracic aorta. First, the image quality of the two different MRA sequences (n = 92) was assessed by visual analysis of vessel contrast (vessel wall versus lumen), sharpness of the vessel wall and artifacts (Fig. 1A). All evaluations were performed independently by two radiologists, each with 5 years of experience in cardiovascular imaging (readers 1 and 2).

For vessel contrast and sharpness of aortic segments, a previously published grading was used [5] with a four-point scale: (1) excellent definition of the aortic wall; (2) good definition of the aortic wall with mild limitations; (3) moderate definition of the aortic wall with substantial limitations; (4) nondiagnostic because of insufficient visualization. The image quality of the thoracic aorta was graded at three segments (the aortic root/ascending aorta, aortic arch and descending aorta) in the source images only. All kinds of artifacts were considered, namely, motion artifacts, signal voids, and radial undersampling artifacts [16]. Second, presence of artifact was rated on a four-point scale: (1) no artifact; (2) mild artifact not interfering with aortic wall definition; (3) moderate artifact degrading aortic wall definition; and (4) severe artifact resulting in nondiagnostic images.

Image quality regarding artifacts was analyzed in the whole study group and after correction for confounding surgical implants (Fig. 1B).

Third, in the subgroup of patients following aortic surgery only, the difference in image quality of the two MRA groups was determined (Fig. 1C). Fourth, in the subset of patients with both MRA sequences, image quality was compared intraindividually (Fig. 1D). Because of the distinct image appearance of each MRA, these sequences could not be blinded.

Additionally, in patients with both MRA acquisitions, any abnormal findings, such as aortic aneurysm (diameter > 4 cm), dissection, intramural hematoma, and penetrating aortic ulcer, were evaluated.

### Quantitative analysis

Signal-to-noise ratio (SNR) was measured in the aortic root and anterior to the body. It was calculated as the signal intensity from the desired region of interest (ROI) divided by the standard deviation of the background noise. Scan time was recorded.

**Fig. 2:**
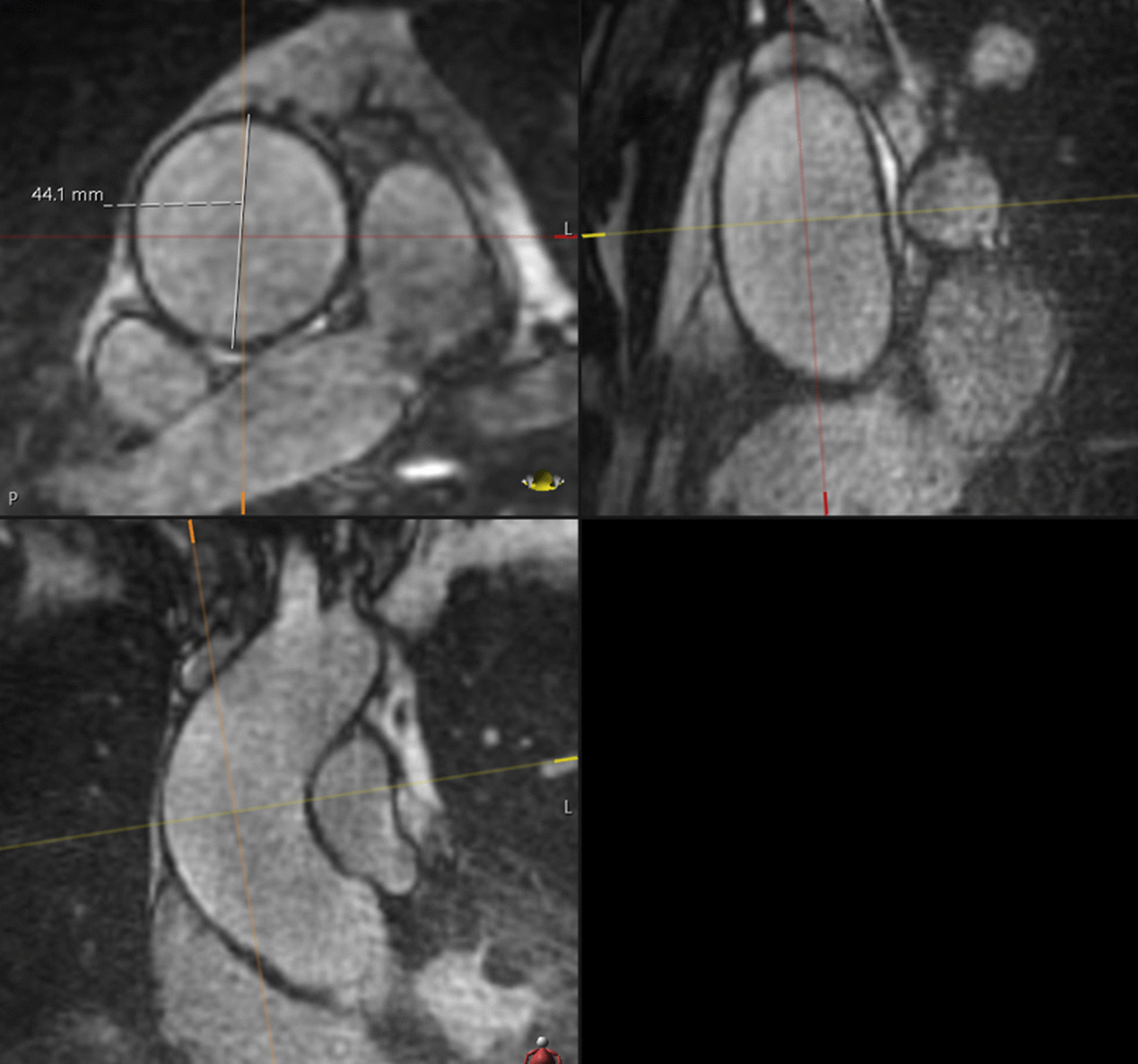
Diameter measurement. For example, the diameter of an aneurysmal aortic segment of the ascending aorta at the level of the right pulmonary artery measured axially, perpendicular to the blood-filled lumen outer wall to the outer wall in multiplanar reformat

In patients with both MRA acquisitions, the largest diameter of aneurysmal aortic segments and the diameter in normal segments, such as the ascending aorta at the level of the right pulmonary artery, the descending aorta and the pulmonary artery, were compared in the two MRA groups. Therefore, axial diameters were measured perpendicular to the blood-filled lumen outer wall to outer wall by one reader in multiplanar reformat (Fig. 2). For diagnosing thoracic aortic aneurysms, age- and sex-matched limits were used [17].

### Statistical analysis

To measure interrater agreement, the weighted Cohen`s kappa coefficient (κ) for categorical data with an ordinal structure was calculated at the three aortic locations including the 95% confidence intervals (CIs) with a bootstrap method and 2000 repetitions.

To test for significant differences between the image quality of the two MRA sequences, 4-point scale ratings were compared with the Mann–Whitney U test. For small sample volumes, Fisher`s exact test was applied. To achieve binary ratings, 4-point scale ratings were recoded as “excellent/good/moderate” (1, 2, and 3) versus “non diagnostic” (4). The CIs for the risk difference were calculated according to the method of Agresti-Caffo [18].

Artifacts are strongly associated with surgical implants such as valve replacements, and sternal cerclages. To avoid confounding factors, we stratified the study population into patients who underwent surgery of the aortic root up to and including the aortic arch and those who were not operated on these regions (Fig. 1). For the analysis of the influence of artifacts on the image quality of both sequences, first, Fisher`s exact test was used for the whole study group, followed by the Mantel–Haenszel formula [19] to address confounding surgical implants. The Mantel–Haenszel method is a technique that generates an estimate of an association between an exposure (different MRA groups/techniques) and an outcome (“good” or “bad” image quality) after adjusting for or taking into account confounding (operation: “yes” or “no”) in categorical data. The method is used with a dichotomous outcome variable and a dichotomous risk factor. The CIs for the risk differences were calculated according to Klingenberg et al. [20].

Quantitative variables, e.g., SNR, and scan time were compared with Student’s t-test or the Wilcoxon rank sum test. P values of 0.05 or less were considered significant. All statistical analyses were performed with the statistical software R (version 3.5.1, R Foundation for Statistical Computing, Vienna, Austria).

## Results

### Participants

After the initial exclusion of patients who received intravascular contrast agents, no further patients were excluded. Altogether, 15 patients (48.4%) in the native-SN-MRA group and 42 patients (68.9%) in the CE-MRA group underwent aortic surgery of the aortic root up to and including the aortic arch (Table 2).

**Table 2:** Distribution of aortic operations in the aortic patient collective

Operation	OP AA/AAr	OP DA	No operation	Total
Native-SN-MRA	15 (48.4%)	3 (9.7%)	13 (42%)	31
CE-MRA	42 (68.9%)	2 (3.3%)	17 (28%)	61

*CE-MRA* contrast-enhanced MRA, *Native-SN-MRA* 3D radial respiratory self-navigated non-contrast-enhanced MRA, *AR* aortic root, *AA* ascending aorta, *AAr* aortic arch, *DA* descending aorta, *OP* operation

In patients with both MRA acquisitions, any pathologic findings, such as aortic aneurysm (diameter greater than 4.0 cm), dissections, intramural hematoma or penetrating ulcer on native-SN-MRA, were confirmed on CE-MRA datasets. Consequently, there were no false positives or negatives on native-SN-MRA, yielding 100% sensitivity, specificity, positive and negative predictive values, and diagnostic accuracy for the detection of aortic disease.

### Qualitative analysis

#### Aortic image quality

The interrater agreement was higher than 0.81 at all aortic locations analyzed in both MRA groups (Table 3a). Because of the high interrater agreement, the results of reader 1 were used for further statistical analysis.

**Table 3:** Results for aortic image quality and artifact properties

a) Weighted Cohen's Kappa							
Location	Native-SN-MRA Kappa (95% CI)	Native-SN-MRA p	CE-MRA Kappa (95% CI)	CE-MRA p			
Ascending aorta	0.917 (0.811; 1)	< 0.001	1 (1;1)	< 0.001			
Aortic arch	0.877 (0.706; 1)	< 0.001	1 (1;1)	< 0.001			
Descending aorta	0.887 (0.746; 1)	< 0.001	0.954 (0.86; 1)	< 0.001			

b) Distribution of overall image quality							
	Excellent %	Good %	Moderate %	Poor %			
*Ascending aorta*							
Radial MRA	48.4	35.5	12.9	3.2			
CE-MRA	9.8	39.3	31.1	19.7			
*Aortic arch*							
Radial MRA	38.7	38.7	9.7	12.9			
CE-MRA	4.9	39.3	45.9	9.8			
*Descending aorta*							
Radial MRA	60	23.3	13.3	3.3			
CE-MRA	19.7	65.6	11.5	3.3			

c) Overall image quality							
	Native-SN-MRA n = 31	CE-MRA n = 61	Risk difference (95% CI)	p (binary)	p (4-point scale)		
*Location*							
Ascending aorta	30 (97%)	49 (80%)	0.17 (0.017; 0.274)	0.05	< 0.001		
Aortic arch	27 (87%)	55 (90%)	-0.3 (0.185; 0.104)	0.73	< 0.001		
Descending aorta	29 (94%)	59 (96%)	-0.02 (-0.114; 0.084)	1	0.005		

d) Artifact properties before and after stratification of the two MRA groups							
	Native-SN-MRA OP yes n = 15	Native-SN-MRA OP no n = 16	CE-MRA OP yes n = 42	CE-MRA OP no n = 19	p (binary)	Risk difference (95% CI)	p (Mantel–Haenszel test)
*Location*							
Ascending aorta	13 (87%)	13 (81%)	16 (38%)	14 (74%)	< 0.001	0.305 (0.097; 0.475)	0.008
Aortic arch	13 (87%)	11 (69%)	16 (38%)	11 (56%)	0.015	0.32 (0.099; 0.501)	0.008
Descending aorta	14 (93%)	11 (69%)	34 (81%)	18 (95%)	0.404	− 0.02 (− 0.19; 0.13)	0.791

e) Patients after aortic surgery							
	Native-SN-MRA n = 15	CE-MRA n = 42	Risk difference (95% CI)	p			
*Location*							
Ascending aorta	13 (87%)	16 (38%)	0.486 (0.206; 0.669)	< 0.001			
Aortic arch	13 (87%)	16 (38%)	0.486 (0.206; 0.669)	< 0.001			
Descending aorta	14 (93%)	34 (81%)	0.124 (− 0.107; 0.281)	0.002			

f) Overall image quality in patients with both MRA acquisitions							
	Native-SN-MRA n = 10	CE-MRA n = 10	Risk difference (95% CI)	p			
*Location*							
Ascending aorta	8 (80%)	5 (50%)	0.3 (− 0.481; 0.313)	0.153			
Aortic arch	8 (80%)	6 (60%)	0.2 (− 0.124; 0.624)	0.099			
Descending aorta	9 (90%)	10 (100%)	− 0.1 (− 0.205; 0.538)	0.348			

*MRA* MR angiography, *CE-MRA* contrast enhanced MRA, *Native-SN-MRA* 3D radial respiratory self-navigated non contrast-enhanced MRA, *SD* standard deviation, *OP* operation

**Fig. 3:**
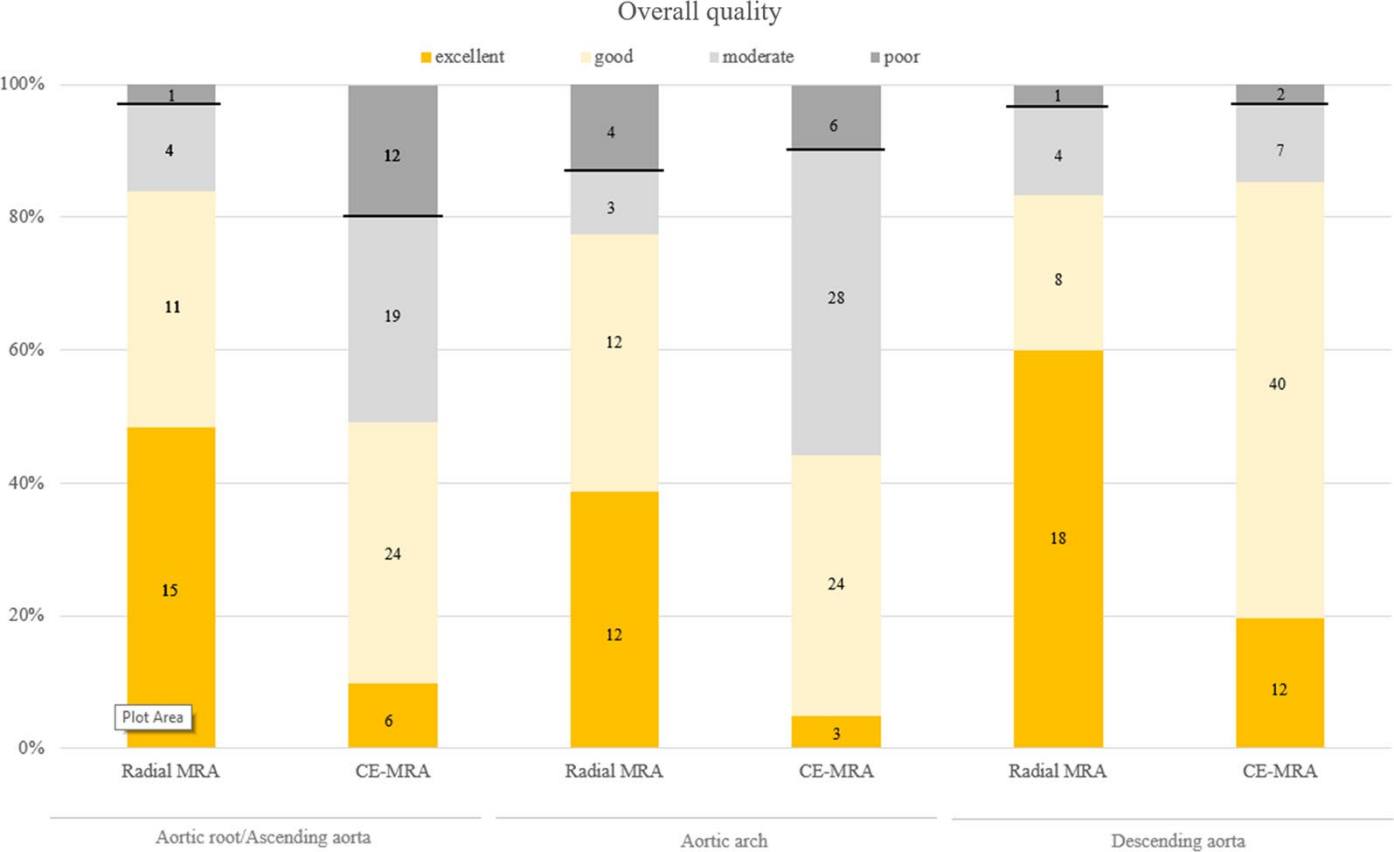
Overall image quality. Comparison of overall image quality for radial self-navigated non-contrast-enhanced magnetic resonance angiography (radial MRA, n = 31) and contrast-enhanced MRA (CE-MRA, n = 61) at the three aortic locations rated as excellent (orange), good (light orange), moderate (light gray) and poor (dark gray). The black line separates the two groups for binary ratings in “excellent/good/moderate” versus “nondiagnostic”. *CE-MRA* contrast-enhanced magnetic resonance angiography

**Fig. 4:**
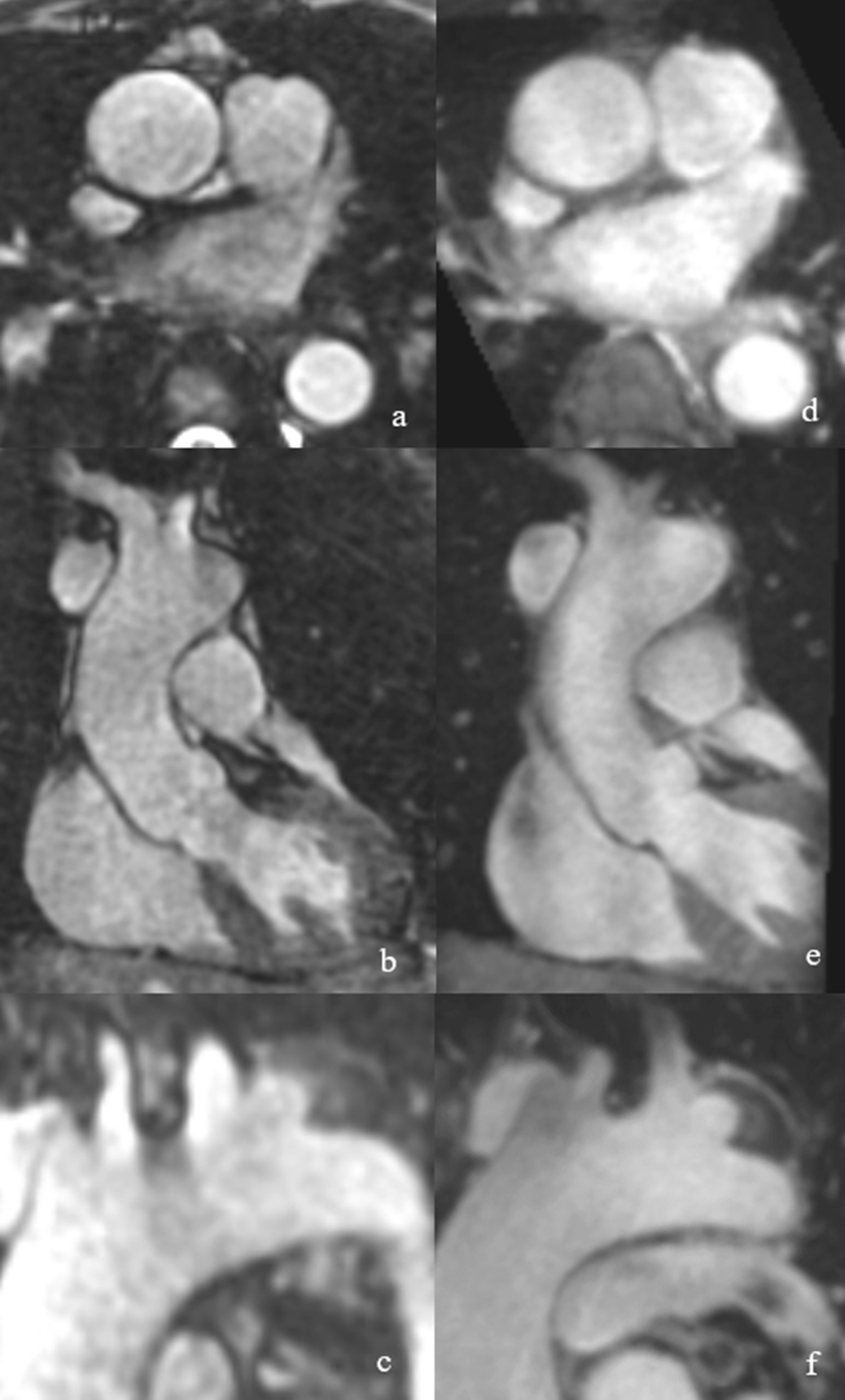
Patient example of overall image quality between native-SN-MRA and CE-MRA. Intraindividual comparison of a 46-year-old female vascular patient demonstrating the overall quality of native-SN-MRA (**a** and **b**) and CE-MRA (**c** and **d**). In native-SN-MRA, the wall of the aortic root/ascending aorta is sharper and better defined (**a** axial and **b** coronal MPR) as in CE-MRA (**c** axial and **d** coronal MPR). *CE-MRA* contrast-enhanced magnetic resonance angiography, *native-SN-MRA* 3D radial respiratory self-navigated non-contrast-enhanced MRA, *MPR* multiplanar reconstruction

More native-SN-MRA acquisitions were in the excellent group than in CE-MRA at all 3 locations (the aortic root/ascending aorta 48.4%, aortic arch 38.7% and descending aorta 60% in native-SN-MRA, and the aortic root/ascending aorta 9.8%, aortic arch 4.9%, and descending aorta 19.7% in CE-MRA). At the aortic root/ascending aorta and aortic arch, fewer native-SN-MRA acquisitions were in the poor image quality group (Table 3b and Figs. 3 and 4). The majority of CE-MRA acquisitions were located in the good group at the aortic root/ascending aorta and descending aorta (39.3% and 65.6%) and in the moderate group at the aortic arch (45.9%) (Table 3b). To achieve diagnostic image quality, “excellent, good, and moderate” were selected as one group in binary ratings (Fig. 3). In this setting, only the aortic root/ascending aorta showed a significant (p = 0.05) high-risk difference of 0.17 between native-SN-MRA and CE-MRA (Table 3c). A significant difference was not found at the aortic arch or descending aorta (p = 0.73 and p = 1, respectively). When using 4-point scale ratings, all three aortic segments reached significance (p < 0.001, p < 0.001, and p = 0.005) (Table 3c) due to the distribution in the subgroups (Table 3b and Fig. 3).

#### Artifact properties

**Fig. 5:**
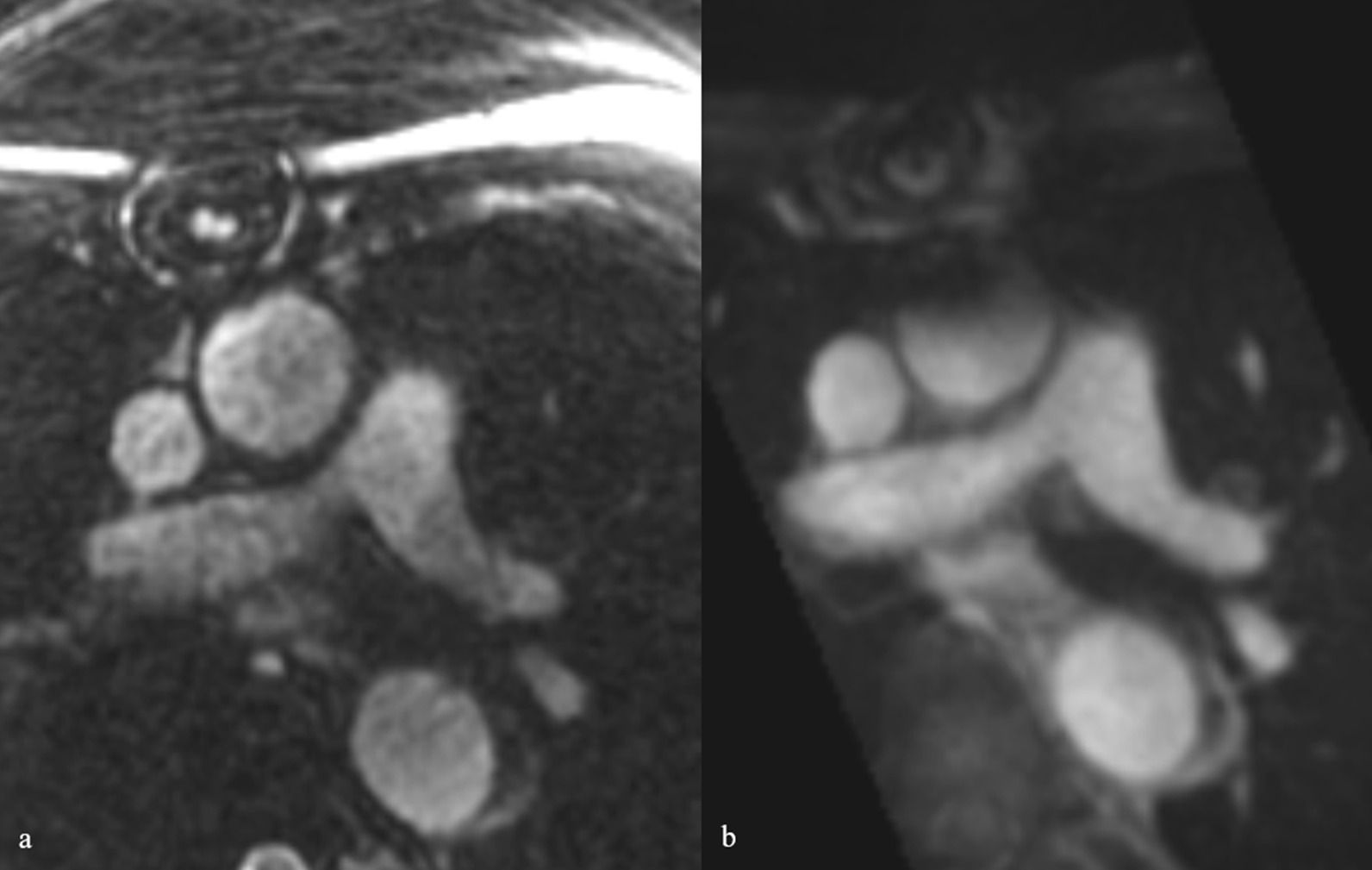
Example of superior image quality between native-SN-MRA and CE-MRA regarding artifacts. Native-SN-MRA (**a**) and CE-MRA (**b**) in a 52-year-old female vascular patient after replacement of the aortic root/ascending aorta and the aortic arch because of aortic dissection type A (Stanford), demonstrating lower susceptibility to artifacts, which was due to sternal cercalges. *CE-MRA* contrast-enhanced magnetic resonance angiography, *native-SN-MRA* 3D radial respiratory self-navigated non-contrast-enhanced MRA

Native-SN-MRA showed significantly fewer artifacts than CE-MRA at the aortic root/ascending aorta and the aortic arch (p < 0.001 and p = 0.015) in the whole study group (Table 3d). Stratifying the two MRA groups into patients who underwent aortic surgery (from the aortic valve up to and including the aortic arch) and those who were not operated on, reduced the confounding of artifacts following surgery, in particular susceptibility-related artifacts. After this correction, significance was still attained at the aortic root/ascending aorta and the aortic arch (both p = 0.08) (Table 3d, Fig. 5). No difference between the two MRA groups was demonstrated at the descending aorta (p = 0.791). The following analysis in the subgroup consisting of only patients who underwent aortic surgery demonstrated superior image quality for native-SN-MRA at all aortic segments analyzed (p < 0.001 the aortic root/ascending aorta and aortic arch and 0.002 the descending aorta) (Table 3e).

#### Intraindividual image quality

In the intraindividual analysis, native-SN-MRA showed a trend towards superior image quality at the aortic root/ascending aorta and the aortic arch (risk differences of 0.3 and 0.2, respectively) without reaching significance (p = 0.15 and 0.09, respectively) (Table 3f).

**Fig. 6:**
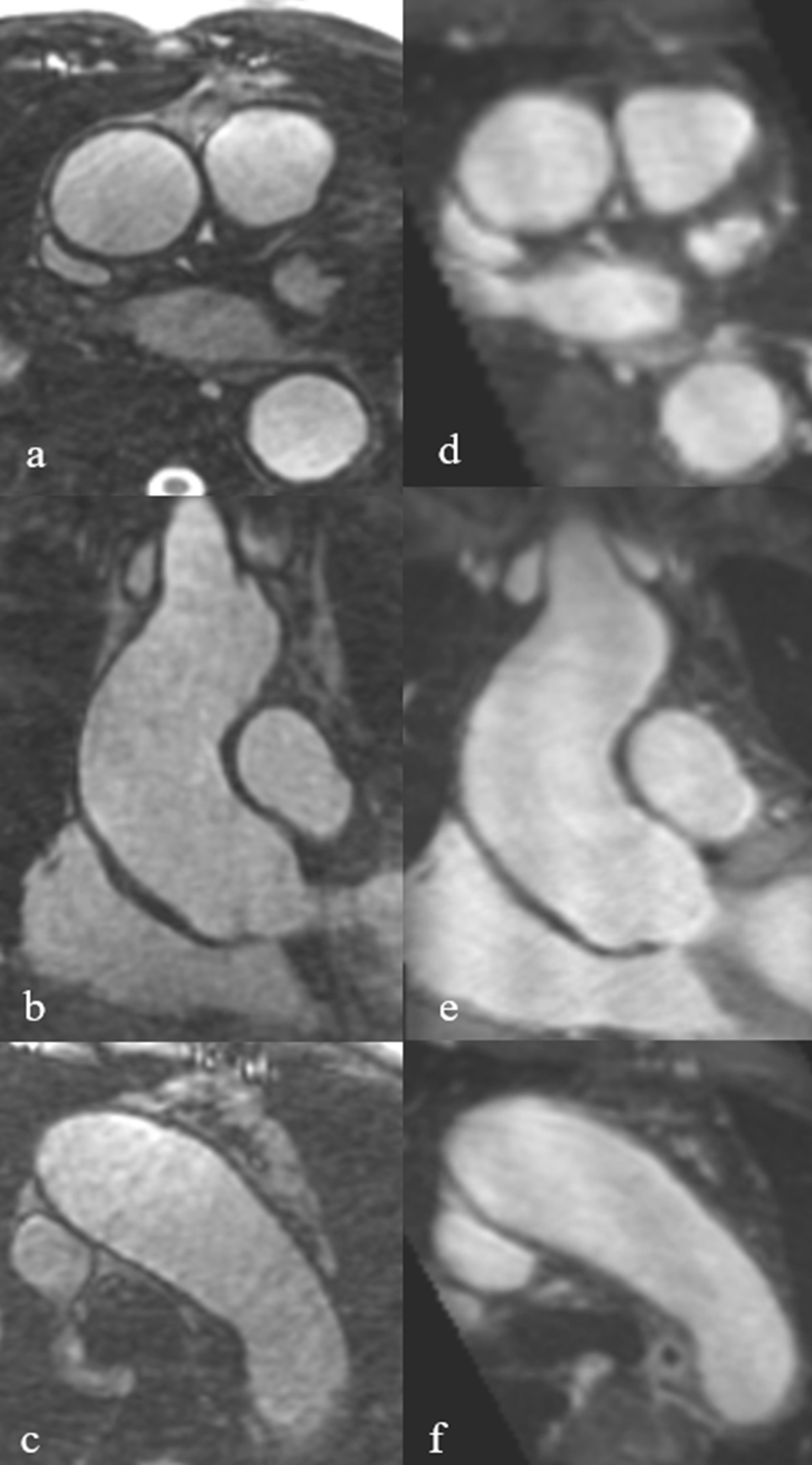
Examples of superior contrast from the vessel wall to the surrounding tissue. Multiplanar reformats of native-SN-MRA (**a**–**c**) and CE-MRA (**d**–**f**) in a 48-year-old male aortic patient indicating superior contrast from the vessel wall of the aortic root/ascending aorta and the aortic arch to the surrounding tissue and fluid, respectively, in native-SN-MRA. *CE-MRA* contrast-enhanced magnetic resonance angiography, *native-SN-MRA* 3D radial respiratory self-navigated non-contrast-enhanced MRA

During evaluation, it was noted that in native-SN-MRA, the contrast from the aortic vessel wall to the extravascular tissue was high as was the contrast to the lumen (see Fig. 6). This finding enabled the visualization of the entire aortic wall. The vessel-extravascular contrast was rated excellent or good in 68% of the ascending aorta, 48% of the aortic arch and 8% of the descending aorta in the native-SN-MRA group. Significance was achieved at all locations analyzed (p < 0.001 for all three locations).

### Quantitative analysis

**Fig. 7:**
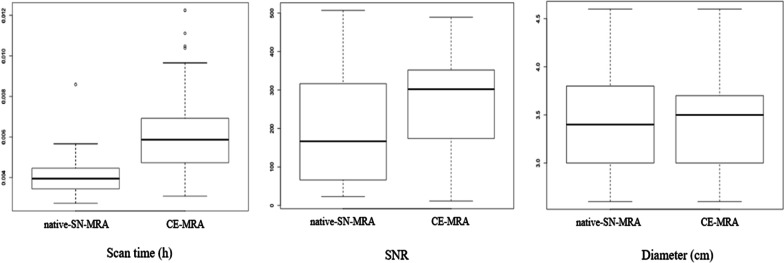
Scan time, SNR and aortic diameter. The scan time was significantly shorter on native-SN-MRA than on conventional high-resolution CE-MRA (p < 0.01). SNR values were significantly higher on CE-MRA (p < 0.02). In the intraindividual comparison of patients with both types of MRA, the measurement of the pathologic and normal diameters did not show a significant difference (p < 0.899). *CE-MRA* contrast-enhanced magnetic resonance angiography, *native-SN-MRA* 3D radial respiratory self-navigated non-contrast-enhanced MRA

As expected, the mean SNR was higher after contrast media administration in the CE-MRA group, with an average improvement of 37.1%. This result was significant (p = 0.016) (Fig. 7).

The scan time was significantly shorter in the native-SN-MRA group, with mean scan times of 05:56 min (SD 01:23 min) and 08:51 min (SD 02:57 min) in the CE-MRA group (p < 0.001) (Fig. 7).

In patients with both MRA acquisitions, the comparison of the diameters of diseased and normal aortic segments showed no significant difference (p = 0.899) between native-SN-MRA (mean 3.4 ± 0.51 cm) and CE-MRA (mean 3.16 ± 0.51 cm) (Fig. 7).

## Discussion

In our study, the native-SN-MRA sequence provided superior image quality of all aortic segments, especially the aortic root/ascending aorta, compared to Cartesian 3D free breathing CE-MRA as the gold standard. This technique provides high spatial resolution unenhanced MRA of the thoracic aorta with superior image quality regarding artifacts. High image quality of the native-SN-MRA sequence was achieved before (p < 0.001 the aortic root/ascending aorta and p = 0.015 the aortic arch) and after correction for confounding aortic surgery (from the aortic valve up to and including the aortic arch) at the aortic root/ascending aorta and the aortic arch (both p < 0.008). First, this result indicates a benefit of the native-SN-MRA sequence in terms of artifacts in general. Additionally, the native-SN-MRA sequence has an advantage in image quality after removing the influence of operations and associated susceptibility artifacts, e.g., on motion artifacts at these two locations as previously reported [9, 15, 21]. In the analysis of the subgroup consisting of only patients who underwent aortic surgery, superior image quality of the native-SN-MRA sequence was noted at all aortic segments analyzed, which was significant (p < 0.001 the aortic root/ascending aorta and the aortic arch, p = 0.002 the descending aorta). A nonsignificant trend was reached at the aortic root/ascending aorta and the aortic arch in the intraindividual comparison in patients with both MRA acquisitions, which was probably due to the small sample volume. In this subgroup, all aortic diseases could be evaluated with high sensitivity, specificity, and diagnostic accuracy in the diagnosis of common and important aortic diseases without the administration of contrast media.

Two major aspects are likely to cause the observed differences in image quality. First, the radial trajectory is less prone to motion artifacts than Cartesian acquisition in the CE-MRA scan [21], an important benefit in imaging regions with pronounced motion as in the aortic root and the ascending aorta. However, radial acquisitions suffer from radial undersampling artifacts that usually appear as streaking artifacts [16, 22]. These artifacts only become more prominent in the outer part of the FOV, the reason why the region of interest (e.g., aortic root) is positioned in the middle of the FOV. Second, bSSFP is known to be prone to off-resonance banding artifacts; however, the signal voids caused by susceptibility effects in the aorta (not in the direct vicinity of the sternal cerclages) were similar between spoiled gradient echo and bSSFP, but a behavior as shown in Fig. 5 was also observed resulting in the statistics as presented in Table 3e. As shown by Scheffler et al. a partial refocusing of T2*-related signal decay can be present depending on the frequency range within a voxel [23]. Based on our observations, we assumed that in the outer vicinity of the cerclages (e.g., in the lumen of the aorta), the frequency range of the off-resonance frequencies is that low, so fewer signal voids can appear in such areas.

Our results are in line with the study group of Haji-Valizadeh et al. [24], where comparable image quality and quantitative results of non-contrast MRA vs. CE-MRA were found. Additionally, in our analysis, overall image quality, especially in motion-prone segments of the thoracic aorta, could be shown to be superior, e.g., the aortic root and the aortic arch, which was not distinguished by the colleagues. This may outcome be due to our 3D radial acquisition compared to their stack-of-stars trajectory.

It was noted that in native-SN-MRA, the contrast from the aortic vessel wall to the extravascular tissue was also high (Fig. 6). This finding enabled the visualization of the entire aortic wall and was probably due to residual fat signal in the native-SN-MRA sequence notwithstanding the fat saturation (Figs. 4 and 6). Since in radial acquisition each signal readout crosses the center of k-space, a CHESS-type prepulse is known to be nonideal [22]. It has already been shown that with improved signal suppression from superfluous tissues such as the chest wall, the self-navigation and therefore image quality could be further improved [25].

The data sets of both MRA acquisitions contained scans with moderate to poor image quality. This inferior image quality was mostly due to motion and susceptibility artifacts, because this study population consisted of patients with aortic pathologies, predominantly those with surgical implants following aortic surgery. Furthermore, CE-MRA appeared blurrier than native-SN-MRA, which may be related to the navigator gating window (6 mm width), centric reordering or the fact that the scan duration was longer than the duration of contrast infusion. However, to obtain a robust image quality of cardiac motion prone segments, the use of ECG-triggering is essential, which again requires the use of a respiration control approach.

Notably, native-SN-MRA presented with a clearly reduced scan time compared to navigator-gated CE-MRA because of the data acceptance rate of 100% [9], an important issue in data efficiency in daily routines. Because of the ECG synchronization and respiration control required, the scan time was in the range of minutes rather than in the range of approximately 20 s as in the “conventional” MRA in the breath-hold technique. Therefore, the flow rate of the contrast agent was rather low in comparison to that of “conventional” MRA. Despite centric k-space reordering, venous signal enhancement was observed depending on the acceptance rate within the first seconds of the acquisition. However, this issue did not affect diagnostics.

The aim of this study was to ensure reliable diameter measurements in the thoracic aorta, an important issue in the follow-up of patients with aortic diseases. Analysis of diameter measurements for the diseased and normal aortic segments revealed no significant difference. This finding suggests that native-SN-MRA has the potential to provide reliable assessment of the severity of aortic dilatation and aneurysms, which is essential in clinical decision-making.

### Limitations

As a limitation of this study, the patient group with both MRA acquisitions was rather small, impeding generalization of the results. Therefore, larger studies are needed in the future. There was no detailed analysis on the image quality of the supra-aortic branches in this study. Since the native-SN-MRA sequence has a rather small isotropic FOV of 25 cm [21], the supra-aortic branches are often located outside the FOV. Further scientific work needs to be done to address this issue. To cope with this demand in daily practice, at the moment, we perform a breath-hold 3D T1-weighted gradient echo pre and post contrast media covering a great volume craniocaudally. The combination of native-SN-MRA and postcontrast MRA allows us to reduce the dose of contrast media by a factor of two, which is currently the case in our protocol. However, the best value would definitely be to avoid contrast agent completely. As a limitation of the native-SN-MRA sequence, it should be noted that diagnostically important vessel wall enhancement in inflammatory vascular pathologies [26], e.g., postoperative infection and autoimmune vasculitis, is not detected with this sequence. In such cases, the application of contrast media is still needed.

## Conclusions

High-resolution 3D radial respiratory SN non-contrast MRA provides superior image quality of the entire thoracic aorta, including segments prone to cardiac motion, the aortic root and the ascending aorta offering a benefit regarding gadolinium safety. As the acquisition time is shorter and because it is gadolinium-free, sequences can also be more readily repeated. Larger studies are needed to confirm the high diagnostic accuracy of this novel native-SN-MRA technique.

## Data Availability

The datasets used and/or analyzed during the current study are available from the corresponding author on reasonable request.

## Abbreviations

bSSFP: Balanced steady state with free precession
CE: Contrast-enhanced
ECG: Electrocardiogram
FOV: Field-of-view
Gd-BOPTA: Gadobenate dimeglumine
MPR: Multiplanar reconstruction
MRA: Magnetic resonance angiography
SN: Self-navigated
SNR: Signal-to-noise ratio
TE: Echo time
TR: Repetition time
OP: Operations
